# Bioconversion of wild *Ipomoea pes-caprae* and *Suaeda fruticosa* biomass: a novel application of thermostable xylanase from *Neobacillus sedimentimangrovi*

**DOI:** 10.1186/s12896-025-00974-6

**Published:** 2025-05-14

**Authors:** Rozina Rashid, Uroosa Ejaz, Wissal Audah Alhilfi, Mohammed Alorabi, Syed Tariq Ali, Muhammad Sohail

**Affiliations:** 1 Department of Microbiology, University of Karachi, Karachi, 75270 Pakistan; 2 Department of Microbiology, University of Balochistan, Quetta, Pakistan; 3 Department of Biotechnology, Faculty of Life Sciences, Shaheed Zulfiqar Ali Bhutto Institute of Science and Technology (SZABIST University), Karachi, Pakistan; 4 Marine Science Centre, University of Basrah, Basrah, Iraq; 5 Department of Biotechnology, College of Science, Taif University, Taif, 21944 Saudi Arabia; 6 Department of Chemistry, University of Karachi, Karachi, 75270 Pakistan

**Keywords:** Antioxidant activity, *Lpomea pes-caprae*, *Neobacillus sedimentimangrovi*, *Suaeda fruticosa*, Saccharification, Xylanase

## Abstract

Biomass from halophytes is considered as a promising chemical feedstock. Its bioconversion to obtain reducing sugars and to concomitantly improve antioxidant potential has been described less frequently. This is the first report describing application of xylanase from *Neobacillus sedimentimangrovi* for the saccharification of *Ipomoea pes-caprae* (IPC) and *Suaeda fruticosa* (SF). In this study, the biomass IPC and SF was separately or co-pretreated by freeze-thaw and 1% H_2_SO_4_. Results showed that significant amount of reducing sugar was obtained by saccharification of acid and freeze-thaw pretreated IPC (44 mg g^− 1^) and freeze-thaw pretreated SF (43 mg g^− 1^). The residues after saccharification were also analyzed for their antioxidant potential where IPC residues exhibited 1.13 folds higher potential than that of SF. Antioxidant potential (83.9%) was obtained when purified xylanase was used for the saccharification of IPC. Moreover, absence of lignin-related peaks in the NMR and IR spectra of the treated substrates indicated efficient delignification. The characteristic peaks of the hemicellulosic fractions in saccharified samples were also disturbed, indicating changes in the crystallinity of the substrates. The SEM images and spectra of the saccharified substrates clearly indicated the degradation of hemicellulosic content by xylanse.

## Introduction

Halophytes thrive in saline environments. The coastal areas of the Sindh and Balochistan provinces in Pakistan host nearly 410 halophyte species. It accounts for approximately 19% of the country’s flora [1, 2]. Some halophytes have also been recognized as medicinal plants, mainly due to their antioxidant potential [3]. The saline habitats of these plants require adaptation to harsh conditions, including redox homeostasis. To protect cells from oxidative damage, these plants sustain control between reactive oxygen species (ROS) synthesis and energy outflow in the antioxidant response, either enzymatically or non-enzymatically [4]. Therefore, halophytes are more likely to produce bioactive compounds with higher antioxidant capacities making them better candidates as therapeutic agents. Qasim et al. [5] surveyed 100 medicinal plants from Pakistan’s coastal regions and found that *Ipomoea pes-caprae* (IPC) and *Suaeda fruticosa* (SF) have high antioxidant potential. IPC, a member of Convolvulaceae family, is widely distributed across tropical and subtropical regions. Various species of the genus *Ipomoea* have long been used in traditional medicine to treat numerous health conditions [6]. In addition, it possesses various bioactive properties, including antioxidant, analgesic, anti-inflammatory, antispasmodic, anticancer, antinociceptive, antihistaminic and insulogenic effects. It is also a valuable source of phytochemical antioxidants, which contribute to its medicinal significance [7]. On the other hand, SF is commonly found in the coastal and inland saline regions of Pakistan as well as in the Saharo-Sindian and Southern Iranian-Turkish areas. It contains bioactive compounds with anti-ophthalmic, hypolipidemic, and hypoglycemic properties [8]. The plant also exhibits superior radical scavenging and reducing powers compared to synthetic antioxidants, likely due to the presence of unique biologically active compounds. These properties distinguish it from other antioxidant-rich species, including herbs, food plants, medicinal plants, and other halophytes [5].

The carbohydrate content of halophytic plants can also be extracted in the form of reducing sugars by the enzyme-mediated saccharification [9]. Saccharification depends upon substrate composition, enzyme loadings, reaction conditions, including pH and temperature [10]. Xylanase plays a vital role in saccharification by breaking down hemicellulose, a major structural component of plant biomass, into fermentable sugars [11]. Many microbial species have been reported for their xylanolytic potential. *Neobacillus sedimentimangrovi* UE25 is a thermophilic bacteria which has been reported to produce many plant cell wall degrading enzymes such as cellulase, xylanase, amylase, pectinase and laccase [12]. The strain UE25 was isolated from crocodile pond of Manghopir, Pakistan. Its thermophilic nature makes it highly suitable for industrial applications where enzyme stability towards high-temperature is required [13]. Previously, Rashid et al. [14] characterized and purified the xylanase from *N. sedimentimangrovi* UE25. However, the use of xylanase from *N. sedimentimangrovi* for saccharification of biomass from halophytes has been scarcely reported.

Although enzyme mediated saccharification appeared as a promising technology, however, pretreatment of biomass is required to improve access of the enzymes to the substrates [9]. Consequently, higher levels of reducing sugars obtained from pretreated biomass can be converted to value-added products. Among chemical pretreatment methods, the use of alkali and acid has been widely described. Dilute acid treatment provides several advantages, including a higher conversion rate of cellulose to reducing sugars and release of fewer inhibitors. Freeze–thaw is a physical pretreatment method used to treat biomass from halophytes [15, 16].

This study holds importance as it was aimed to saccharify the pretreated biomass from halophytes using xylanase of thermophilic bacterium, *N. sedimentimangrovi*. Changes in halophytic plant biomass due to pretreatment and saccharification process were studied by Scanning Electron Microscopy, Fourier Transform Infrared Spectroscopy and Nuclear Magnetic Resonance Spectroscopy. Moreover, the saccharified residues were analyzed to study antioxidant potential by studying scavenging activity.

## Materials and methods

### Selection of raw material

Four halophytic plants including IPC (KUH-96778), SF (KUH-98975), *Phragmites karka*, PK (KUH-94112) and *Halopyrum mucronatum*, HM (HM; KUH-94253) were obtained from the Institute of Sustainable Halophyte Utilization University of Karachi. SF, IPC, HM and PK contained 21%, 17%, 28.67% and 29% hemicellulose, respectively. While, lignin content in SF, IPC, HM and PK was 4.67%, 5.33%, 5% and 10.33%, respectively. The plants were identified by Dr Salman Gulzar and submitted to the Karachi University Herbarium (KUH). All the substrates were powdered to the size of 100 µ.

### Procurement of xylanase

Crude enzyme preparation from *N. sedimentimangrovi* UE25 [17] and purified xylanase [14] were obtained from the Department of Microbiology, University of Karachi. Whole genome sequencing (GenBank accession number JAJODE000000000.1) [13] revealed identity of the strain UE25 as *N. sedimentimangrovi* (that was initially reported as *Bacillus aestuarii*).

### Acidic pretreatment of the halophytic substrate

Acidic pretreatment was performed by following the method of Guo et al. [18]. Plant biomass (1 g) was mixed with 20 mL of 1% sulfuric acid and autoclaved at 121^o^C for 1 h. Residues were then filtered, and the slurry was washed with water until excessive acid was removed. Residues were dried at 60^o^C until constant mass and used as acid treated biomass.

### Freeze-thaw pretreatment of the halophytic substrate

The pretreatment by freeze-thaw method was performed according to the method of Smichi et al. [16]. Plant biomass was kept in a freezer at -20 °C for 24 h and then thawed directly at 100 °C in a water bath for 15 min followed by filtration. The process of freezing and thawing was repeated three times. The sample was kept at 60 °C in a hot air oven until constant mass and used as freeze-thaw treated biomass.

### Acidic and freeze-thaw pretreatment of the halophytic substrate

At first, acid pretreatment was applied as given above but with a slight modification by reducing the amount of sulfuric acid (10 mL) to minimize the use of chemical and to observe the effect of reduced amount of acid in the combined pretreatment. After acidic pretreatment, the material was pretreated by freeze-thaw method as described earlier. Sample was used as acid and freeze thaw treated plant biomass.

### Saccharification of the halophytic substrate

Saccharification of the substrates was initiated with a standardized xylanase preparation (either purified or crude) as 10 U in the final reaction volume of 100 mL containing 50mM Sodium citrate buffer (pH 5) containing 1 g of the biomass from halophyte and 0.2 g sodium azide. The reaction mixture was incubated for 24 h in a shaking incubator (150 rpm) at 60^o^C. Aliquots were collected at 0 h and 24 h. Reducing sugars in the hydrolysate were detected by the assay as proposed by Miller [17]. A 25 µL aliquot of hydrolysate was mixed with an equal volume of sodium citrate buffer (50 mM, pH 4.8), followed by the addition of 150 µL of DNS reagent. The mixture was boiled for 5 min, cooled on ice, and diluted with 720 µL of distilled water. The absorbance was measured at 540 nm, and the concentration of reducing sugars was determined by comparing the OD_540_ of the sample with that of the standard glucose solutions [19]. The left over saccharified residues were used to analyze antioxidant potentials.

### Preparation of sample extracts for antioxidant analysis

The saccharified residues were vortexed for 1 h in 10 mL methanol to achieve a concentration of 15, 30, 45 and 60 mg mL^− 1^ (Table 1). The tubes were kept at room temperature for 24 h. Afterwards, mixture was centrifuged for 10 min at 3000 rpm and supernatant was saved to perform scavenging activity test [20].

**Table 1:** Initial screening for saccharification of *Saueda fruticose* (SF), *Ipomea pes-caprae* (IPC), *Halopyrum mucronatum* (HM) and *Phragmites karka* (PK). Residues from halophytes (either treated or untreated) were subjected to crude or purified Xylanase from *Neobacillus sendimentimangrovi* UE25 for 24 h and reducing sugars were estimated

Substrate	Pretreatment method	Reducing sugars (mg g^− 1^)*	
		Crude xylanase	Purified xylanase
SF	Untreated	29	25
	Acid	0	0
	Freeze-thaw	43	24
	Acid + freeze thaw	0	0
IPC	Untreated	38	34
	Acid	0	0
	Freeze-thaw	27	37
	Acid + freeze-thaw	44	38
HM	Untreated	35	12
	Acid	24	24
	Freeze-thaw	24	23
	Acid + freeze-thaw	23	0
PK	Untreated	25	23
	Acid	0	0
	Freeze-thaw	23	0
	Acid + freeze-thaw	22	24

*Insignificant standard deviation

### Antioxidant analysis of halophytes

Antioxidant potential of halophytic biomass was evaluated using 2,2-diphenyl-1-picryl-hydrazyl (DPPH) free radical scavenging test [20]. Briefly, the stock solution was prepared by adding 33.9 mg of DPPH in 100 mL methanol. Previously prepared concentrations of the samples were mixed with methanolic extracts (1 mL each) and kept in dark for 30 min. Absorbance values were noted at 517 nm. The percentage of scavenging activity was calculated as follows:

$$\%\: {\bf{Scavenging}}\,{\bf{activity}} = \,{{Abs\,of\,control - Abs\,of\,sample } \over {Abs\,of\,control}}\, \times 100$$

### Examination of structural changes in IPC and SF

The samples of both IPC and SF were analyzed by scanning electron microscopy to observe any changes in the structure after pretreatment and saccharification process. The samples were dried at 60^o^C for 48 h and images were captured at 40 µ scale at a voltage of 10 kV. The thin films of the samples were processed for IR spectroscopy and the bands were recorded in the region 400–4000 cm^− 1^ on JASCO FTIR-4200. NMR spectroscopy of the samples was performed at 700 MHz in DMSO-d6 solutions using Bruker (AV-700 MHz) spectrometer. The ppm (δ) depicts the sign of chemical shift to the signal related to DMSO d6.

### Statistical analysis

Origin Pro 8 was used to analyze the data. Mean and standard deviation were calculated. Mean value having standard deviation < 20% was presented. Regression analysis was performed to analyze antioxidant data.

## Results and discussion

### Saccharification of biomass from different halophytic plants

Pretreatment of plant biomass is needed prior to its utilization for microbial fermentation and enzymatic conversion. Brown et al. [21] reported a higher yield of glucose from pretreated *Salicornia bigelovii* biomass in comparison to untreated substrate. Acid pretreatment of halophytic biomass has been also reported earlier, where acid pretreated *Juncus maritimes* was used for the saccharification by a mixture of fungal xylanase and cellulase [16]. In this study, saccharification of untreated and treated halophytic plants, HM and PK through purified and crude xylanase produce similar amounts of reducing sugars (~ 25 mg g^− 1^) (Table 1). However, the impact of pretreatment to IPC and SF was evident on the saccharification process. Freeze-thaw treatment appeared to be a better pretreatment strategy for SF as the saccharification of this pretreated substrate by crude xylanase produced the highest titer of 43 mg g^− 1^ (~ 18%) reducing sugars. However, saccharification of the same substrate by the purified xylanase yielded 24 mg g^− 1^ (~ 10%) of reducing sugar. The freeze-thaw procedure has been reported to degrade plant cell wall and to increase the availability of polysaccharides to hydrolyzing enzymes [22]. The saccharification of acid-freeze thawed pretreated IPC by crude xylanase yielded 44 mg g^− 1^ (corresponding to ~ 23% saccharification), whereas, purified xylanase-mediated saccharification of the same substrate resulted in the release of 38 mg g^− 1^ (~ 20%) of reducing sugars. Acid-freeze thaw was found as a promising pretreatment method for this substrate; the strategy reduced the use of acid to half of its quantity. The release of more reducing sugars by crude xylanase mediated saccharification of IPC may be attributed to the presence of various modulators present in the crude preparation that enhanced the enzymatic activity [15]. Previously, only 2.8 and 0.05 mg g^− 1^ reducing sugars were obtained after the saccharification of *Panicum antidotale* and *Halopyrum mucronatum*, respectively [9].

Moreover, acidic pretreatment resulted in no release of reducing sugars in few experimental conditions (Table 1) which might be because of formation of by-products which can result in inactivity of enzyme. Acidic treatment can also result in structural modifications of substrate making xylanase binding ineffective [23, 24]. Considering the preliminary saccharification results, IPC pretreated with acid and freeze-thaw method and SF pretreated by freeze-thaw process were selected for further experiments.

Nonetheless, reducing sugars produced by saccharification have significant potential for various industrial applications, particularly in biofuel production, pharmaceuticals, and the food industry. In biofuel production, reducing sugars can serve as fermentable substrates for microbial fermentation, leading to the generation of bioethanol and other biofuels [25].

### Estimation of antioxidant activity by DPPH assay

The polyphenolic components with antioxidants capabilities are found in certain plant extracts and have the ability to donate electrons and generate free radicals [26]. Salt-resistant plants generally exhibit antioxidant potential [27, 28]. In this study, the substrates left after saccharification were analyzed for antioxidant activity through 2,2-diphenyl-1-picryl hydrazyl (DPPH) assay. Various workers have found a connection between the antioxidant properties of phenols and polyphenols to their capacity to scavenge DPPH radicals [6]. The DPPH assay is a widely accepted and reliable method to evaluate antioxidant potential due to its ability to directly measure free radical scavenging activity. Furthermore, DPPH assay sufficiently represents overall antioxidant activity [29, 30].

After saccharification with purified xylanase, the IPC residue yielded a maximum free radial scavenging potential of 83.9% that was 1.08 folds higher than that of unsaccharified IPC (77.7%). The antioxidant potential of the residue saccharified with crude xylanase was found to be 79.22%. Scavenging potential of 76.92% was observed for SF saccharified with crude xylanase that was 1.03 folds higher than the unsaccharified SF (74.4%). Moreover, the scavenging capabilities of SF hydrolyzed by purified xylanase (74.27%) and unsaccharified SF did not differ greatly. The results of saccharified residues of IPC and SF revealed that antioxidant capacity of methanol extracts increased with increasing substrate concentration (Table 2). Regression analysis showed significance of the result with p-value > 0.0001 indicating that scavenging activity significantly increases with substrate concentration. Earlier, Sujatha et al. [31] reported about the antioxidant potential of IPC. Whereas, Oueslati et al. [32] and Chekroun-Bechlaghem et al. [33] investigated the antioxidant capacity of SF. A comparative account on antioxidant capacity of SF with a remedial herb *Juglans regia* also concluded SF as a better substrate [8] mainly due to the presence of higher polyphenol content. Moreover, unsaccharified plant biomass resulted in less scavenging activity in contrast to saccharified sample (Table 2). Enzymatic saccharification breaks down complex carbohydrates and cell wall structure which can result in the release of phenolic compounds, flavonoids, and other antioxidants from the lignocellulosic matrix [34]. In the pharmaceutical sector, antioxidant-rich residues can be utilized in the formulation of nutraceuticals or therapeutic compounds owing to their bioactive properties [35]. Additionally, in the food industry, these residues can be incorporated into functional foods, natural preservatives, or dietary supplements to enhance their nutritional value [36].

**Table 2:** Determination of antioxidant activity by 2, 2-diphenyl-1-picryl hydrazyl radical (DPPH)

Samples	Substrate^a^	Concentration of substrate (mg mL^− 1^)			
		60	45	30	15
		Scavenging activity (%)*			
1	SF-crude^a^	76.92	55.33	49.66	46.33
2	SF-purified^a^	74.27	49.48	35.64	32.56
3	SF unsaccharified	74.44	56.35	40.82	36.48
4	IPC crude^a^	79.22	69.74	66.41	49.23
5	IPC purified^a^	83.9	71.38	70.33	46.41
6	IPC unsaccharified	77.71	65.12	64.35	54.61

^a^*Saueda fruticosa* (SF) saccharified with either crude xylanase (SF-crude) or purified xylanase (SF-purified) and *Ipomea pes-caprae* (IPC) either saccharified with crude xylanase (IPC-crude) or purified xylanase (IPC-xylanase). The results were compared with the substrate without enzymatic saccharification

*Insignificant standard deviation

### Scanning electron microscopy of IPC and SF

**Fig. 1:**
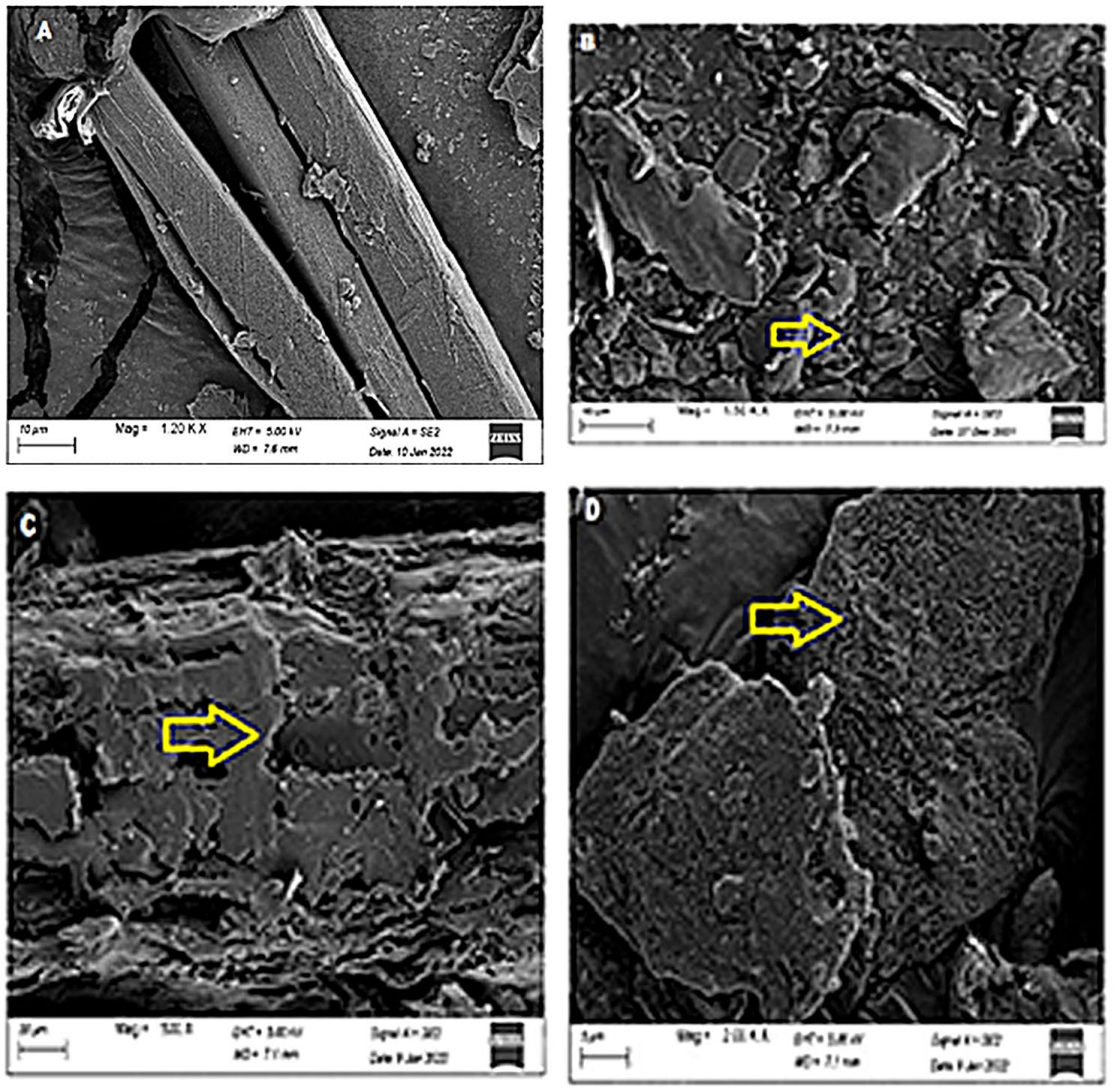
Scanning electron micrographs of *Ipomea pes-caprae*** A**) untreated, **B**) acid-freeze-thawed pretreated, **C**) saccharified by crude xylanase and **D**) saccharified by purified xylanase. Arrow indicated the destructed structure

**Fig. 2:**
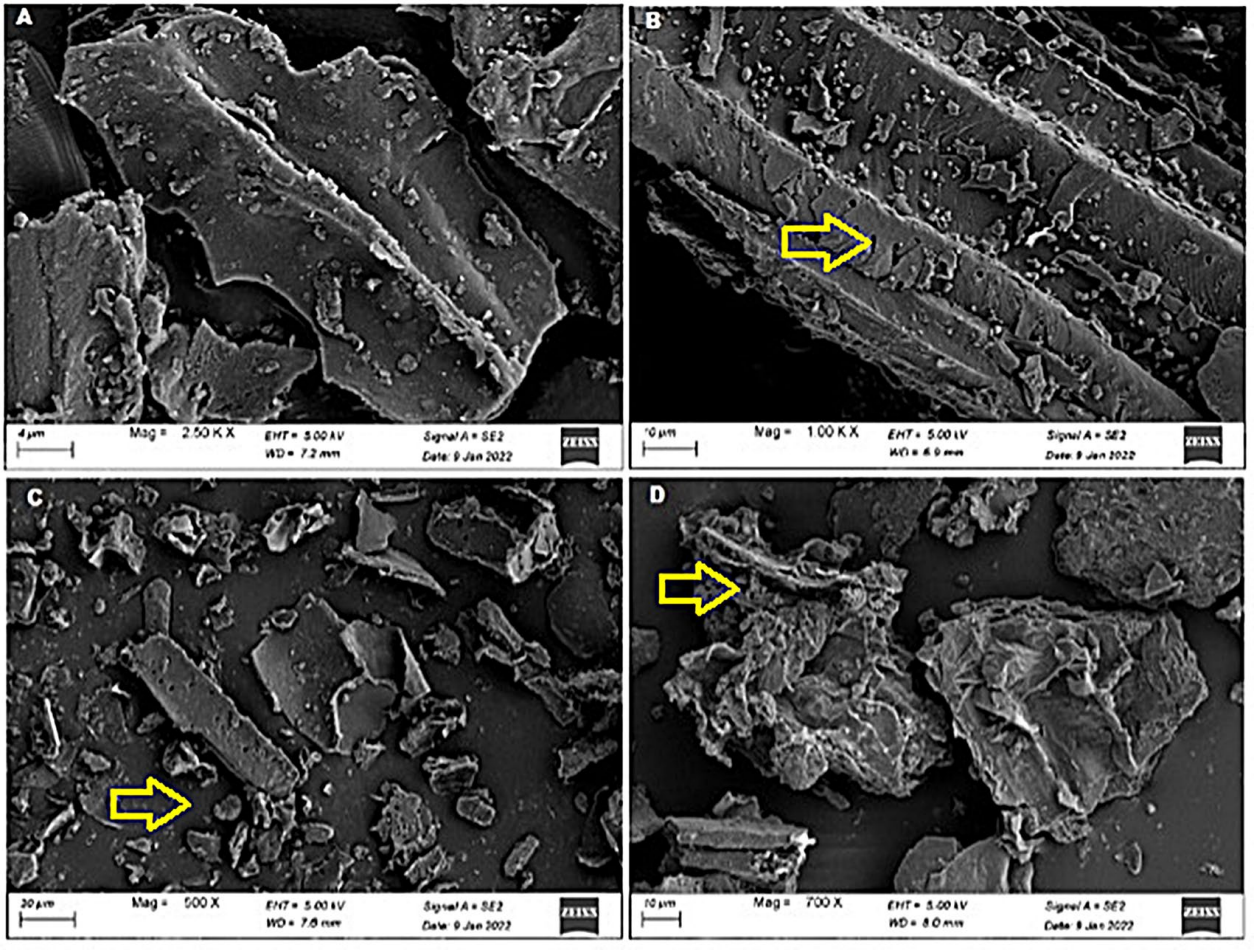
Scanning electron micrographs of *Suaeda fruticosa* biomass **A**) untreated, **B**) freeze-thawed pretreated, **C**) saccharified by crude xylanase and **D**) saccharified by purified xylanase. Arrow indicated the destructed structure

The dense structures of untreated IPC and SF were examined using the SEM images (Figs. 1 and 2). Substantial disruption and damaged surfaces of freeze-thawed SF and acid-freeze-thawed pretreated IPC were observed (Figs. 1 and 2). The changes were analogous to the observations by Canilha et al. [37] where dilute sulfuric acid pretreatment caused separation of the pith of the substrate from fibers. Disruption in SF and IPC structures indicated the utilization of xylan by the activity of crude xylanase (Figs. 1 and 2). A ruptured and porous structural matrix was present in purified xylanase-mediated saccharified substrate of IPC and SF. Earlier, Ansari et al. [9] observed loosening of the structural matrix and porous surface due to the effect of an enzyme cocktail on the wild PK biomass.

### FTIR analysis of IPC residue

**Fig. 3:**
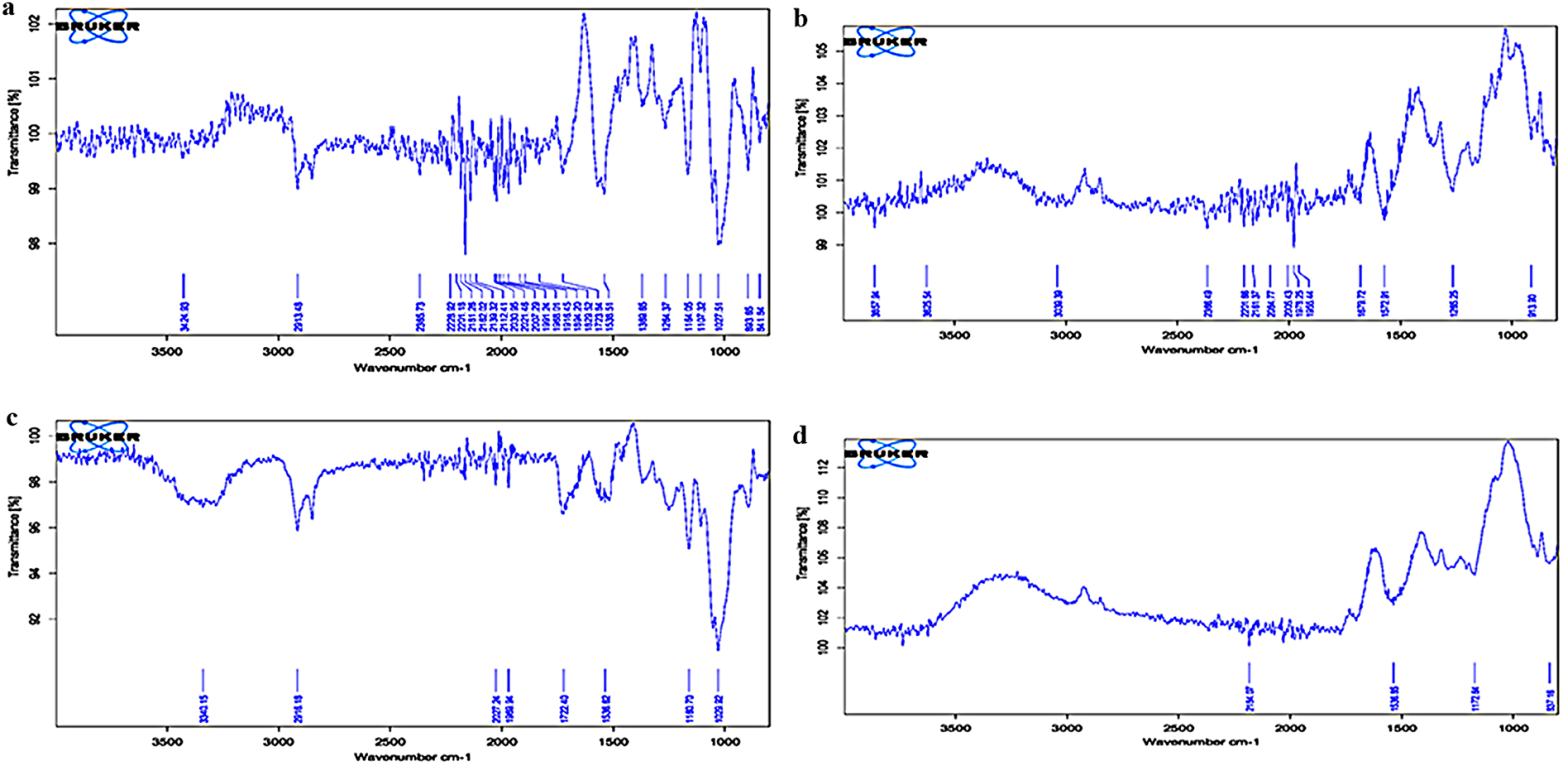
FTIR spectra of *Ipomea pes-caprae* biomass **A**) untreated, **B**) acid-freeze-thawed pretreated, **C**) saccharified by purified xylanase and **D**) saccharified by crude xylanase. Mentioned values corresponds to plant cell wall different regions

FTIR spectra of the IPC residues (Fig. 3) prior to pretreatment, after pretreatment and after saccharification using purified or crude xylanase showed major differences in the structure (Table 3). The absence of several lignin-related peaks and differences in the region of 1700–1720 cm^− 1^ corresponded to the alteration in the composition of lignin attributable to the effect of pretreatment. In the pretreated samples, FTIR spectra highlighted -CH_2_ and -CH asymmetrical stretching related to cellulose at 2918.46 cm^− 1^ and the amended regions at 1109.77 cm^− 1^ and 1161.31 cm^− 1^ by hemicelluloses which showed the presence of hemicellulose and cellulose [17].

**Table 3:** FTIR spectral data for *Ipomea pes-caprae* (IPC) with corresponding peak assignment

Substrate	Peaks	Description
Untreated	893	β-glycosidic linkages between the xylose units
	1027	C-O stretching in lignin; C_6_-O_6_H stretching in cellulose
	1164	aromatic C-H in plane deformation in guaiacyl ring
	1264	G ring breathing with carboxyl stretching
	1370	phenolic OH and aliphatic C-H in methyl group. Attributed to cellulose
	1538	aromatic skeletal vibration in lignin
	1700–1720	beta-keto structures in lignin
	2365	CH_2_ and CH symmetrical stretching in cellulose
	2913	C-H stretching in lignin
	3424	O-H stretching in lignin
Pretreated	1265	G ring breathing with carboxyl stretching
	2366	CH_2_ and CH symmetrical stretching in cellulose
	913	C_6_-O_6_H stretching in hemicellulose
Saccharified with purified xylanase	1029	C_6_-O_6_H stretching dominant in cellulose
	1160	C-O-C stretching in cellulose and xylan
	1722	C = O stretching in acetyl group and carboxylic acid in hemicellulose
	2918	C-H stretching in cellulose
Saccharified with crude xylanase	1172	aromatic C-H deformation in guaiacyl ring
	2000–2400	asymmetric C ≡ C stretching

The vibrations at 1600 cm^− 1^ were due to the stretching of COO- in xylan [38]. Saccharification of the sample by purified and crude xylanase resulted in notable changes in the structure of IPC. The modifications between the regions 1030-1160.40 and 1350–1450 cm^− 1^ presented the effective hydrolysis of xylan. The regions related to C_2_-H mannosyl residues at ~ 875 cm^− 1^ and anomeric region of β-glycosidic linkages at 895 cm^− 1^ were also affected by the saccharification of the biomass, which indicated the utilization of xylan content of IPC [38, 39]. Similar to previous findings, alteration in the area 1043 cm^− 1^ [40] and a peak at 2918 cm^− 1^ [41] confirmed the effect of xylanase on the biomass used here. The decrease in hemicellulose content of IPC due the action of xylanase was evident by considering the peak at 895 cm^− 1^ [42] linked with anomeric region at glycosidic linkages and the peak at 875 cm^− 1^ connected to C_2_-H mannosyl residues. Additionally, the absence of a signal at 913 cm^− 1^ and other hemicellulose-related peaks demonstrated the consumption of xylan in the saccharification process.

### FTIR analysis of Suaeda fruticosa (SF) residue

**Fig. 4:**
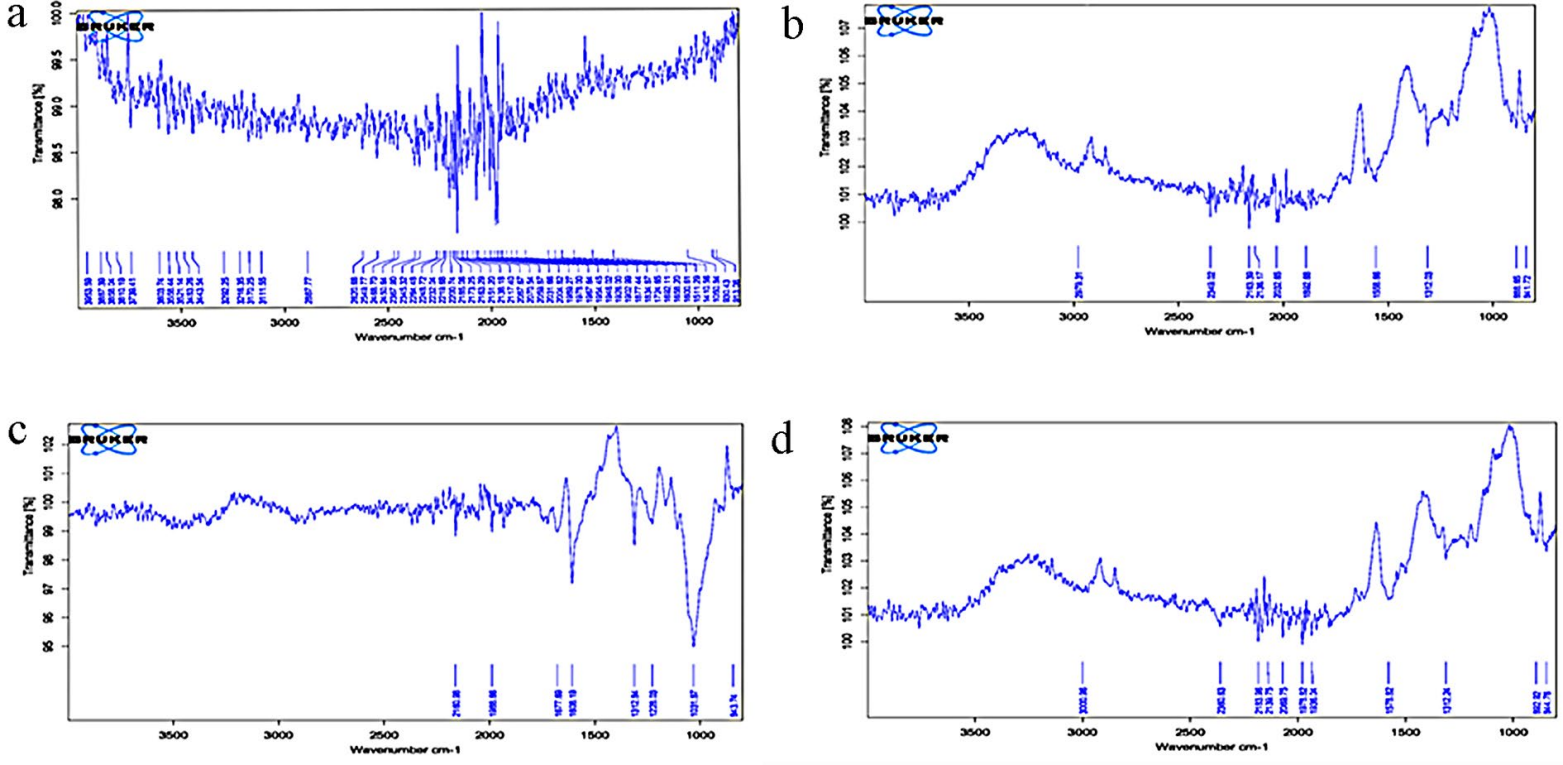
FTIR spectra of *Suaeda fruticosa* biomass **A**) untreated, **B**) freeze-thawed pretreated, **C**) saccharified by purified xylanase and **D**) saccharified by crude xylanase. Mentioned values corresponds to plant cell wall different regions

FTIR analysis of SF (Fig. 4) revealed structural changes in untreated, pretreated, and saccharified residues using purified and crude xylanase. The details of the typical or altered peaks are given in Table 4. Visible differences were observed between untreated and pretreated samples. The absence of characteristic bands related to lignin, including aromatic skeletal vibration (at 1600–1690 cm^− 1^), C = O stretching (at 1690–1727 cm^− 1^), syringyl ring (at 1500–1600 cm^− 1^) [43], functional groups (at 1511 cm^− 1^) [44], and C-H deformation (at 1420 cm^− 1^) [45] indicated effective removal of lignin. The preservation of hemicellulosic content during pretreatment was evident by considering the stretched vibration at region 1422–1454 cm^− 1^ [38]. The utilization of hemicellulose content and structural change as a result of purified and crude xylanase-mediated saccharification was indicated by an extensive stretching vibration at 3403 cm^− 1^ of the -OH groups and C-H stretching vibrations at 2923 and 2850 cm^− 1^ [46]. Changes in the region of 1312 cm^− 1^ (attributed to hemicellulose) and absence of hemicellulose-related peaks in both the samples further confirmed the hydrolysis of hemicellulose content during saccharification.

**Table 4:** FTIR spectral data for *Suaeda fruticosa* (SF) with corresponding peak assignment

Substrate	Peaks	Description
Untreated	1420	C-H deformation
	1600–1690	aromatic skeletal vibration in lignin
	1511	functional group in lignin
	2800–3000	asymmetric, symmetric methyl and methylene CH cellulose group
	3430–3443	characteristics of OH group in lignin
	3000–3700	hydrogen bonded (O–H) stretching
Pretreated	1312–1315	OH stretching in cellulose
	1690	C = O stretching
	1727	C = O stretching
	1500	syringyl ring
Saccharified with purified xylanase	1031	C-H deformation of secondary alcohols and aliphatic ethersaromatic C-H deformation
	1312	recognized as hemicellulose
	1608	COO-stretching in hemicellulose
Saccharified with crude xylanase	892	aromatic vibration at β-glycosidic linkage in hemicellulose
	1312–1315	OH stretching in cellulose

### NMR spectroscopy of Ipomea pes-caprae (IPC) residue

**Fig. 5:**
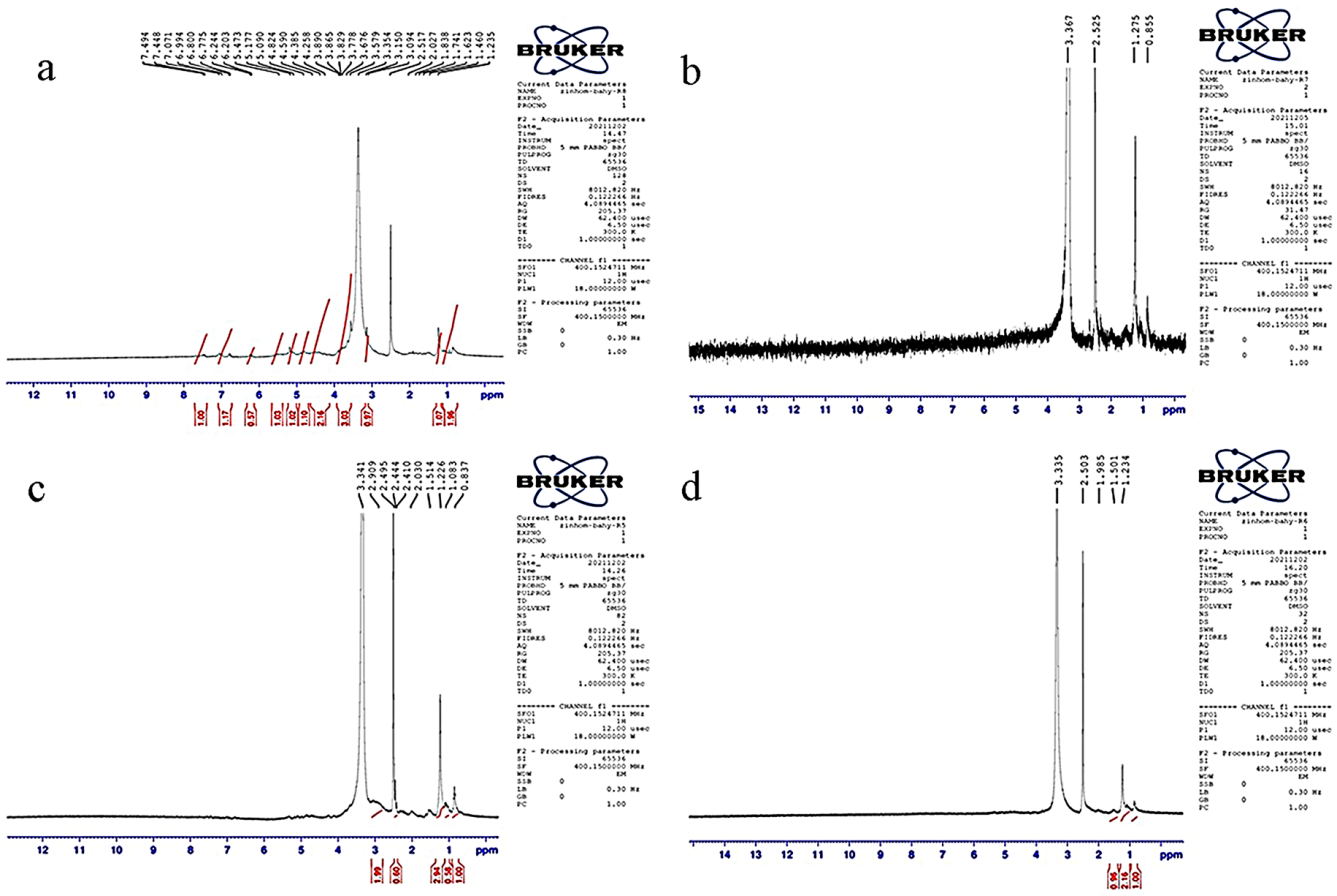
NMR spectra of *Ipomea pes-caprae* biomass **A**) untreated **B**) acid-freeze-thawed pretreated **C**) saccharified by purified xylanase and **D**) saccharified by crude xylanase. Mentioned values corresponds to plant cell wall different regions

The 1 H Nuclear Magnetic Resonance (NMR) spectra of the untreated, pretreated, and saccharified IPC with purified, and crude xylanase showed noticeable patterns (Fig. 5). The pretreated biomass did not exhibit any resonance between 6.5 and 6.9 ppm confirming the removal of lignin. While in the untreated IPC, the signals in the region between 6.7 and 7.2 ppm represented the aromatic protons in “Guaiacyl-Syringyl (G-S) lignin” and the signals at 0.8 to 1.4 manifested the aliphatic parts of the lignin fragment [39, 47]. Signals in the proton dimension between 3.1 and 4.5 ppm were depicted because they are typical of arabinose and xylose residues. Particularly, Methoxyl protons (-OCH3), an indication of the G: S ratio, caused a strong signal between 3.8 ppm and 3.7 ppm [48]. The presence of xylan was indicated by the resonance at 3.3 ppm for H-3 in the 1–4 connected β-D-xylose residues and at 5.1 ppm due to the anomeric protons in xylose at the reducing end terminal [47]. The chemical shifts at 3.5 and 3.8 ppm indicated H-2 and H-3 of the 4-OMe-α-d-Glucuronopyranosyl acetate residues, respectively. Signals specific to hemicellulose in the ring protons area at 3.5, 3.6 and 3.3 ppm were found in the untreated IPC [46], whereas pretreatment and saccharification of IPC was evident by the signals at 3.3 ppm [48, 49]. A prominent signal of DMSO (2.5 ppm) was observed in all samples.

### NMR spectral analysis of Suaeda fruticosa (SF) residue

**Fig. 6:**
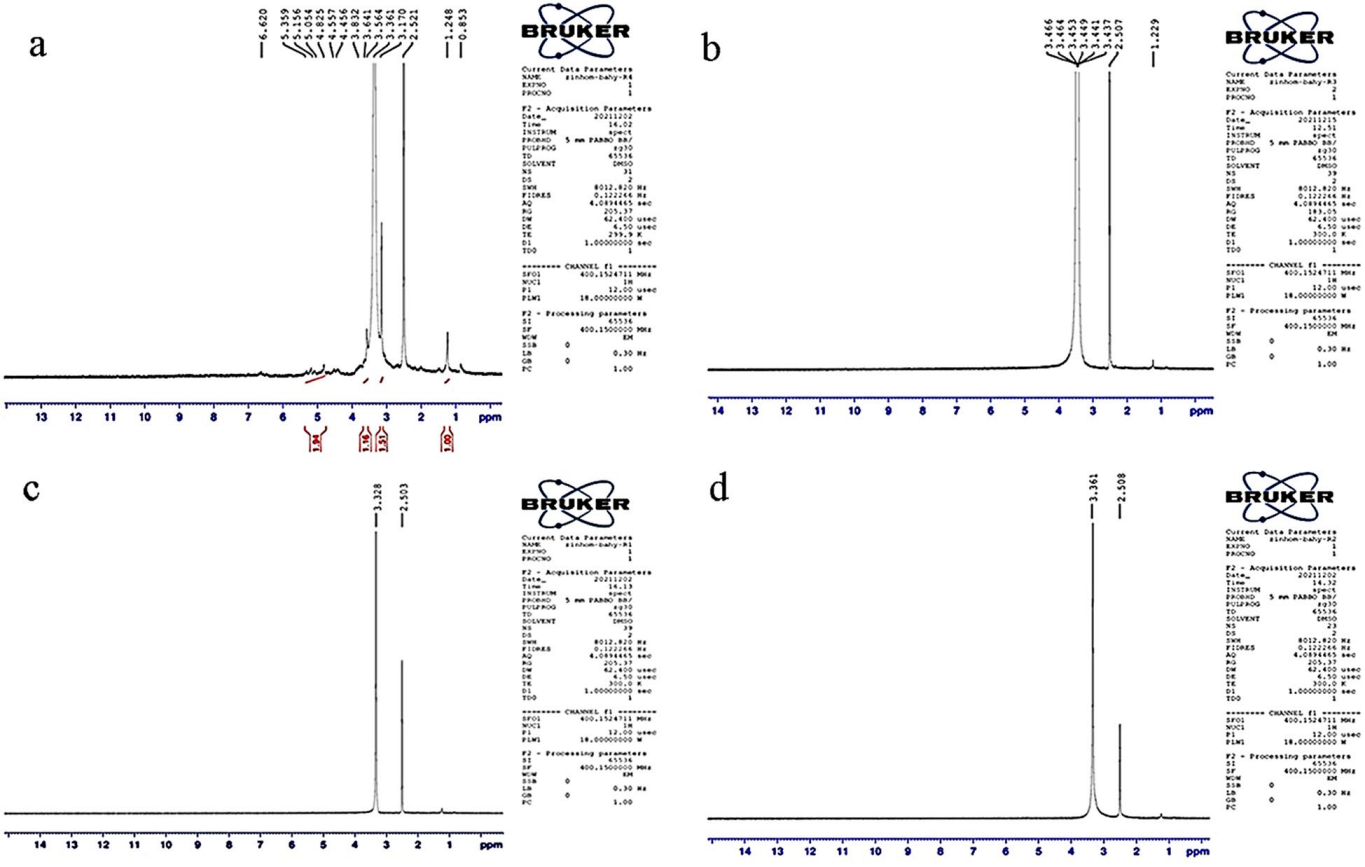
NMR spectra of *Suaeda fruticosa* biomass **A**) untreated **B**) freeze-thawed pretreated, **C**) saccharified by purified xylanase and **D**) saccharified by crude xylanase. Mentioned values corresponds to plant cell wall different regions

NMR spectroscopic analysis of untreated, pretreated, and saccharified residues of SF with purified and crude xylanase confirmed the efficiency of the processes investigated in this study (Fig. 6). The pretreated sample did not exhibit signals of aromatic protons at 6.6 ppm and aliphatic parts of the lignin molecules at 0.8 ppm. The presence of hemicellulose was evident in the anomeric region with the resonance at 5.33 ppm represented terminal α-arabinose residues connected to O-3. Moreover, the signals related to xylan mainly found in the aromatic region at δ 4.90–4.30 ppm and ring proton region at δ 4.50–3.00 ppm. The signal at 4.55 ppm corresponding to β-d-xylose substituted at the C-3 position in the untreated SF confirmed the intact structure of xylan. A characteristic signal of H-2 involved in (1→4)-β-d-Xyl*p*-2-*O*-(4-OMe-α-d-Glc*p*A) linkage was detected at δ 3.44 in the pretreated SF [49]. Considerable changes were observed in the structure after the saccharification of SF, either by purified or crude xylanase. A strong signal at 2.5 ppm in all samples represented the protons in DMSO.

## Conclusion

This study showed that the biomass from *Ipomoea pes-caprae* and *Suaeda fruticosa* can be saccharified after pretreatment to obtain reducing sugars. Moreover, the leftover saccharified residues can also be used as a source of antioxidants and hence, the work promotes zero-waste strategy. The thermophilic bacterium *Neobacillus sedimentimangrovi* UE25 was found as a promising source of xylanase which hydrolyzed biomass from halophytes effectively. The future work necessitates scalability of pretreatment methods, identification of oligosaccharides produced during saccharification and the molecules responsible for antioxidant potential of the saccharified residues.

## Data Availability

The data and materials can be obtained from the first author upon a reasonable request.
